# The Synergism of Human *Lactobacillaceae* and Inulin Decrease Hyperglycemia via Regulating the Composition of Gut Microbiota and Metabolic Profiles in db/db Mice

**DOI:** 10.4014/jmb.2304.04039

**Published:** 2023-08-21

**Authors:** Peifan Li, Tong Tong, Yusong Wu, Xin Zhou, Michael Zhang, Jia Liu, Yongxin She, Zuming Li, Yongli Li

**Affiliations:** 1 College of Biochemical Engineering, Beijing Union University, Beijing, 100023, P.R. China; 2 Department of Physics and Astronomy, University of Manitoba, Winnipeg, MB R3T 2N2, Canada; 3 Sino Canada health engineering research institute, Hefei, P.R. China; 4 Internal Trade Food Science and Technology (Beijing) Co., Ltd, Beijing, 102209, P.R. China; 5 Institute of Quality Standard and Testing Technology for Agro-Products, Chinese Academy of Agricultural Science, Beijing, P.R. China; 6 Henan Provincial People’s Hospital, People’s Hospital of Zhengzhou University, Zhengzhou, P.R. China

**Keywords:** Human *Lactobacillaceae*, type 2 diabetes mellitus, inulin, gut microbiota, metabolomics

## Abstract

This study aimed to evaluate the effects of *Limosilactobacillus fermentum* and *Lactiplantibacillus plantarum* isolated from human feces coordinating with inulin on the composition of gut microbiota and metabolic profiles in db/db mice. These supplements were administered to db/db mice for 12 weeks. The results showed that the *Lactobacillaceae* coordinating with inulin group (LI) exhibited lower fasting blood glucose levels than the model control group (MC). Additionally, LI was found to enhance colon tissue and increase the levels of short-chain fatty acids. 16S rRNA sequencing revealed that the abundance of *Corynebacterium* and *Proteus*, which were significantly increased in the MC group compared with NC group, were significantly decreased by the treatment of LI that also restored the key genera of the *Lachnospiraceae_NK4A136_group*, *Lachnoclostridium*, *Ruminococcus_gnavus_group*, *Desulfovibrio*, and *Lachnospiraceae_UCG-006*. Untargeted metabolomics analysis showed that lotaustralin, 5-hydroxyindoleacetic acid, and 13(S)-HpODE were increased while L-phenylalanine and L-tryptophan were decreased in the MC group compared with the NC group. However, the intervention of LI reversed the levels of these metabolites in the intestine. Correlation analysis revealed that *Lachnoclostridium* and *Ruminococcus_gnavus_group* were negatively correlated with 5-hydroxyindoleacetic acid and 13(S)-HpODE, but positively correlated with L-tryptophan. 13(S)-HpODE was involved in the "linoleic acid metabolism". L-tryptophan and 5-hydroxyindoleacetic acid were involved in "tryptophan metabolism" and "serotonergic synapse". These findings suggest that LI may alleviate type 2 diabetes symptoms by modulating the abundance of *Ruminococcus_gnavus_group* and *Lachnoclostridium* to regulate the pathways of "linoleic acid metabolism", "serotonergic synapse", and" tryptophan metabolism". Our results provide new insights into prevention and treatment of type 2 diabetes.

## Introduction

Type 2 diabetes mellitus (T2DM) is a metabolic disorder characterized by fasting and postprandial hyperglycemia as well as relative insulin insensitivity [1]. T2DM is the result of complex genetic environment interaction, which is related not only to human genes but also to the gut microbiota [2]. Gut microbiota is one of the most important factors affecting human health [3]. There is rising evidence that dietary compounds, particularly probiotics and prebiotics, can improve the function of the gut microbiota of T2DM [4]. Therefore, there is an urgent need for dietary intervention techniques that can alter the gut microbiota to control T2DM.

Thousands of different microbial species make up the gut microbiota, which is a complex symbiotic ecosystem and an important component of gastrointestinal homeostasis, contributing to many important physiological processes in the host [2, 5]. Gut dysbiosis is linked to metabolic endotoxemia and a broken gut barrier, which can cause obesity, chronic low-grade inflammation, insulin resistance, and T2DM [6, 7]. Short-chain fatty acids (SCFAs) that are produced by gut microbiota via carbohydrate fermentation may have beneficial effects on the host. These SCFAs can defend against invading microorganisms and enhance intestinal barrier function by promoting epithelial cell growth and inherent response to epithelial cell injury [8].

Probiotics are live microorganisms that confer health benefits to the host when consumed in adequate amounts and have been investigated as a potential method for preventing or treating T2DM [9, 10]. Probiotics are generally safe for human consumption and act through the microbiota modulation [11]. There is increasing evidence that *Lactiplantibacillus plantarum* (*L. plantarum*) may have beneficial effects on human health, including improving chronic metabolic diseases such as diabetes, obesity, and non-alcoholic fatty liver disease by regulating the production of short-chain fatty acids, antioxidant enzyme activity, and the balance of gut microbial communities [12, 13]. It has been reported that *L. plantarum* SHY130 can ameliorate hyperglycemia and insulin resistance in high-fat diet (HFD)/streptozotocin-induced diabetic mice by modulating the enteroinsular axis [14]. It has also been found that *Limosilactobacillus fermentum* (*L. fermentum*) MG4295 regulates insulin and gluconeogenesis pathways to anti-hyperglycemic activity in an HFD and sugar-water-induced mouse model [15]. To date, most studies have focused on the anti-hyperglycemic effects of one or two strains. However, microbes have different functions in the body, and combining various strains may result in combined benefits of the different individual microbes. A study has shown that combination of *L. plantarum* CGMCC1.1880 and *L. fermentum* CGMCC1.557 significantly reduced the inflammatory response in HFD mice. Furthermore, the improvement achieved with the combined strains was significantly superior to that of a single strain administered at the same dosage [16].

Inulin (INU) is a fructose-based polysaccharide carbohydrate and a type of soluble dietary fiber obtained from Jerusalem artichokes and chicory plants, which is used as a dietary prebiotic and is not digested and absorbed by the human gastrointestinal tract [17]. However, INU can be fermented by the gut microbiota, leading to a reduction of chronic inflammation and endotoxins by modulating the gut microbiota, and may improve insulin resistance in T2DM mice [18]. It has been reported that supplementation of *L. plantarum* and inulin could improve the gut microbial composition as well as reduce the levels of inflammatory cytokines in T2DM rats [19]. In addition, INU can ameliorate metabolic disorders in high-fat diet rats [20] and may increase glycogen synthesis and facilitate glucose transportation by regulating the PI3K/Akt pathway in T2DM rats [21]. However, the effect of *L. plantarum* and *L. fermentum* coordinating with INU on T2DM has not been reported. Metabolomics has been employed to identify potential biomarkers and elucidate the effects of dietary supplementation on T2DM [22]. However, the contribution of metabolomic changes induced by *Lactobacillaceae* and INU to T2DM treatment remains unclear.

In this study, 16S rRNA gene sequencing and ultra-high-performance liquid chromatography coupled with quadrupole time-of-flight mass spectrometry (UPHLC-Q-TOF/MS) were used to evaluate intestinal metabolomic biomarkers and gut microbiota, to determine the mechanism for reducing hypoglycemic effects of human-derived *Lactobacillaceae* and inulin. We also determined the levels of fasting blood glucose (FBG), and short chain fatty acids (SCFAs) in feces, and conducted Spearman’s correlation to identify the close relationship between gut microbiota and metabolites.

## Materials and Methods

### Study Design and Animal Experiments

Six-week-old db/db (C57BLKS/JNju) mice and wild-type (wt) mice were bought from the JiangSu Gempharmatech Co., Ltd. The animal experiments were conducted in accordance with the guidelines of the Animal Welfare Committee of Beijing Union University. The protocol was approved by the Animal Welfare Committee of Beijing Union University (No.2020-05). A temperature controlled (21 ± 1°C) environment with a 12 h light/12 h dark cycle was provided to each mouse in individual cages. Mice had unrestricted access to food and liquids. After acclimation, twelve wt mice served as normal controls (NC) and thirty-six db/db mice were divided into three groups at random (12 mice per group), as follows: T2DM + *L. plantarum* ZLT22, ZLT25 + *L. fermentum* ZLT11, ZLT305 each at 10^9^ CFU/ml (L); T2DM + *L. plantarum* ZLT22, ZLT25 + *L. fermentum* ZLT11, ZLT305 each at 10^9^ CFU/ml+ Inulin at 5% addition (LI); and T2DM model control group (MC). The L and LI groups were treated by oral gavage once daily at 10 milliliters per kilogram of body weight for 12-weeks. The NC and MC groups were administered the same amount of saline solution per day by oral gavage for 12-weeks.

### Fasting Blood Glucose (FBG)

Blood samples were obtained from the mice by pricking their tails with a sterilized needle, squeezing the tails, and allowing the blood to drip onto glucose test strips. The FBG levels were measured using a rapid glucometer (Roche, Switzerland).

### Histopathological Analysis

Colon tissues were removed from the fixative solution (10% formalin saline for 24 h), washed with water and dehydrated using various concentrations of ethanol. The tissues were then embedded in paraffin, sectioned into thicknesses of 5 μm, stained with Hematoxylin and Eosin, and photographed under a microscope.

### SCFA Content in Cecum

Cecum contents of the four groups were collected at the end of the last gavage. After 50 mg of cecum contents were transferred into a centrifuge tube, 1 ml of 50% methanol aqueous solution was added, and mixing by vortexing for 30 min, then centrifuged for 5 min (4°C, 12,000 rmp). After mixed with 250 mmol/l of 3-Nitrophenylhydrazine hydrochloride at a ratio of 1:1 (v/v), the supernatant was analyzed using an UPLC i-Class system connected to a Xevo TQ-S (triple quadrupole MS/MS) Mass Spectrometer (Waters, UK) to measure SCFA.

### 16S rRNA Sequencing

Microbial community genomic DNA was extracted from all fecal samples using the DNeasy PowerSoil Pro Kit (Qiagen, Germany) according to the manufacturer’s instructions. Concentration and integrity of extracted DNA were checked on 1% agarosegel electrophoresis and determined with NanoDrop 2000 UV- visspectrophotometer (ThermoScientific, USA). The DNA was amplified for the V3-V4 variable region of 16S rRNA genes using primer pairs: 338F (5'-ACTCCTACGGGAGGCAGCAG-3') and 806R (5'-GGACTACHVGGGTWTCTAAT-3') by 9700 PCR thermocycler (ABI, USA). The PCR amplicons were pyro-sequenced on the Illumina MiSeq PE250 platform (USA). The raw data were filtered by QIIME (v1.9.1). The original DNA fragment's paired-end reads were merged with the FLASH (v1.2.11). USEARCH (v11) classified the high-quality reads into operational taxonomic units (OTUs) with a 97% threshold. The taxonomy of OTUs was performed using the Ribosomal Database Project (RDP, v2.11) classifier algorithm, and was used with the SILVA 16S rRNA database for comparison, using a confidence threshold of 70%. Alpha diversity analysis included Shannon. We used unweighted UniFrac distance for Principal Coordinates Analysis (PCoA) and non-metric multidimensional scaling (NMDS) analysis in the beta index analysis.

### Metabolite Extraction

The cecum contents samples were precisely weighed to 50 mg and transferred into 2 ml centrifuge tubes, followed by the addition of a single 6 mm grinding bead. Subsequently, 400 μl of a solution composed of methanol/water (4:1, v/v) and containing 0.02 mg/ml of the internal standard L-2-chlorophenyl alanine was added to each tube. The mixture was then subjected to frozen tissue grinding for 6 min at a temperature of -10°C and a frequency of 50 Hz, followed by low-temperature ultrasonic extraction for 30 min at 5°C and 40 kHz. Afterward, the samples were centrifuged at 13,000 ×*g* and 4°C for 5 min, and the supernatants were collected for UHPLC-MC analysis. Additionally, 20 μl of supernatant was pipetted from each sample and mixed to serve as quality control samples.

### Metabolite Analysis

The samples were analyzed using an UHPLC-MS detector (ExionLC AD System -Triple TOF 5600+, AB Sciex, USA) with an ACQUITY UPLC HSS T3 column (100 mm × 2.1 mm i.d.,1.8 μm; Waters, USA). The column was thermostatically controlled at 40°C and the flow rate was set at 0.4 ml/min. The mobile phases consisted of A (95%water + 5% acetonitrile with 0.1% HCOOH, v/v) and B (47.5% acetonitrile + 47.5% isopropanol + 5% water with 0.1% HCOOH,v/v). The solvent gradient in volumetric ratios was set as follows: 0-0.5 min 0-0% B, 0.5-2.5 min 0%-25% B, 2.5-9 min 25%-100% B, 9-13 min 100% B (total 16 min run).

Data acquisition was performed in the full scan mode (*m/z* range from 50 to 1,000). The source temperature was set to 550°C. The following parameters were used: 50 psi ion source gas, 30 psi curtain Gas, 80 V declustering potential, 40 ± 20 eV collision energy, 5,000 V ionspray voltage floating (+), and -4,000 V ionspray voltage floating (-). Full scan mode was used in the mass range of 50 -1,000 *m/z*.

Raw data from UHPLC-MS were preprocessed with missing value recoding and normalization. The data matrix was analyzed by principal component analysis (PCA) and orthogonal partial least-squared discriminant analysis (OPLS-DA) using ropls (R packages, V1.6.2) software. Additionally, the R2X, R2Y, and Q2 parameters were used to evaluate the model. Metabolite markers that potentially change between different experimental groups were selected according to the variable importance in the projection (VIP > 1) from the partial OPLS-DA, *p* value < 0.05, and fold change (FC) >1 or FC < 1. The significance of the metabolite intensity differences among different groups was calculated using Student’s t-test. The Kyoto Encyclopedia of Genes and Genomes (KEGG)(http://www.kegg.com/) was used as biochemical database to identify potential markers and metabolic pathways.

### Statistical Analysis

Statistical analysis was performed with SPSS 26 and Excel 2019. The results were presented as mean ± SD (standard deviation). FBG, SCFAs, the relative abundance of OTUs, and the Shannon index, as well as the relative abundance of differential metabolites and gut microbiota were all compared among the four groups using one-way analysis of variance (ANOVA). *p* < 0.05 was considered statistically significant.

## Results

### Physical Characteristics

**Fig. 1:**
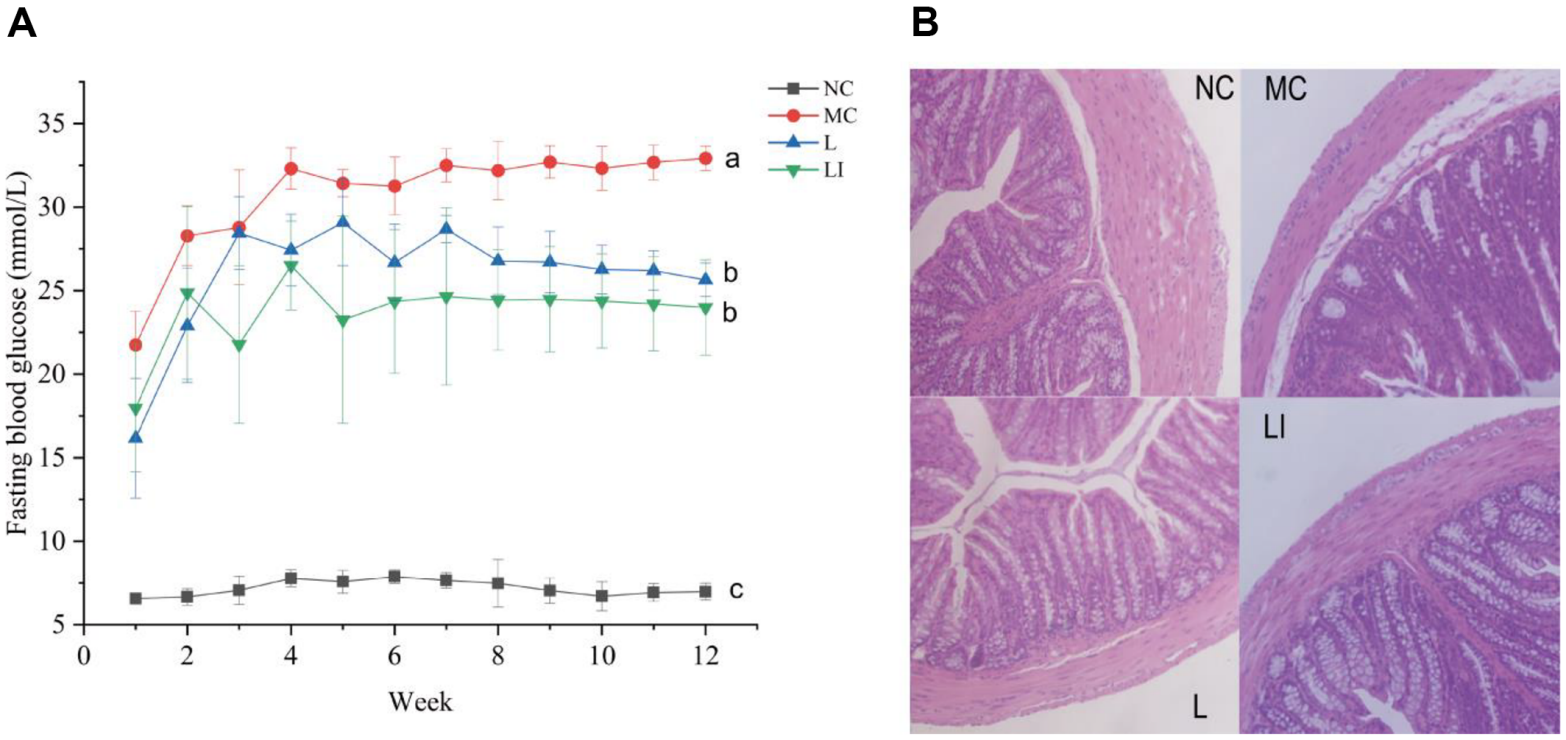
Effects of different treatment groups on blood glucose and colonization in T2DM mice. (**A**) Fasting blood glucose from 1 to 12 week. (**B**) Representative colonic HE staining results of NC, MC, L and LI groups.

The changes in FBG levels are shown in Fig. 1A. Initial FBG levels showed no significant difference among MC, L, and LI groups. After the 12 weeks of administration, the FBG levels in the L and LI groups were significantly decreased compared to the MC group, but there was no significant difference between the L and LI groups. The result of the morphological analysis is shown in Fig. 1B. In the NC group, the structure of the colon was well-organized and showed no pathology. Compared with the NC group, the colon wall in the MC group became thinner, the surface of the colon appeared fractured, massive cells were shed, and there was significant inflammatory infiltrate injury in the colon. However, there were more intact crypt structures and less inflammatory infiltration in the L and LI groups than those in the MC group. Additionally, the colon wall was found to be thicker in the LI group than in the L group.

### Cecal SCFA Content

According to Table 1, the T2DM mice had significantly lower content of acetic acid, propionic acid, butyric acid, and total SCFAs than the NC mice did. The content of acetic and butyric acid in the LI and L groups was significantly higher than that in the MC group, while the content of acetic acid and propionic acid in the LI group was significantly higher than that in the L group. In addition, the content of propionic acid in the LI group increased significantly compared to the MC group, but there was no significant difference between the MC and L groups. These results show that LI exerted better effects on the production of SCFAs than L did.

### Changes in Gut Microbiota

**Fig. 2:**
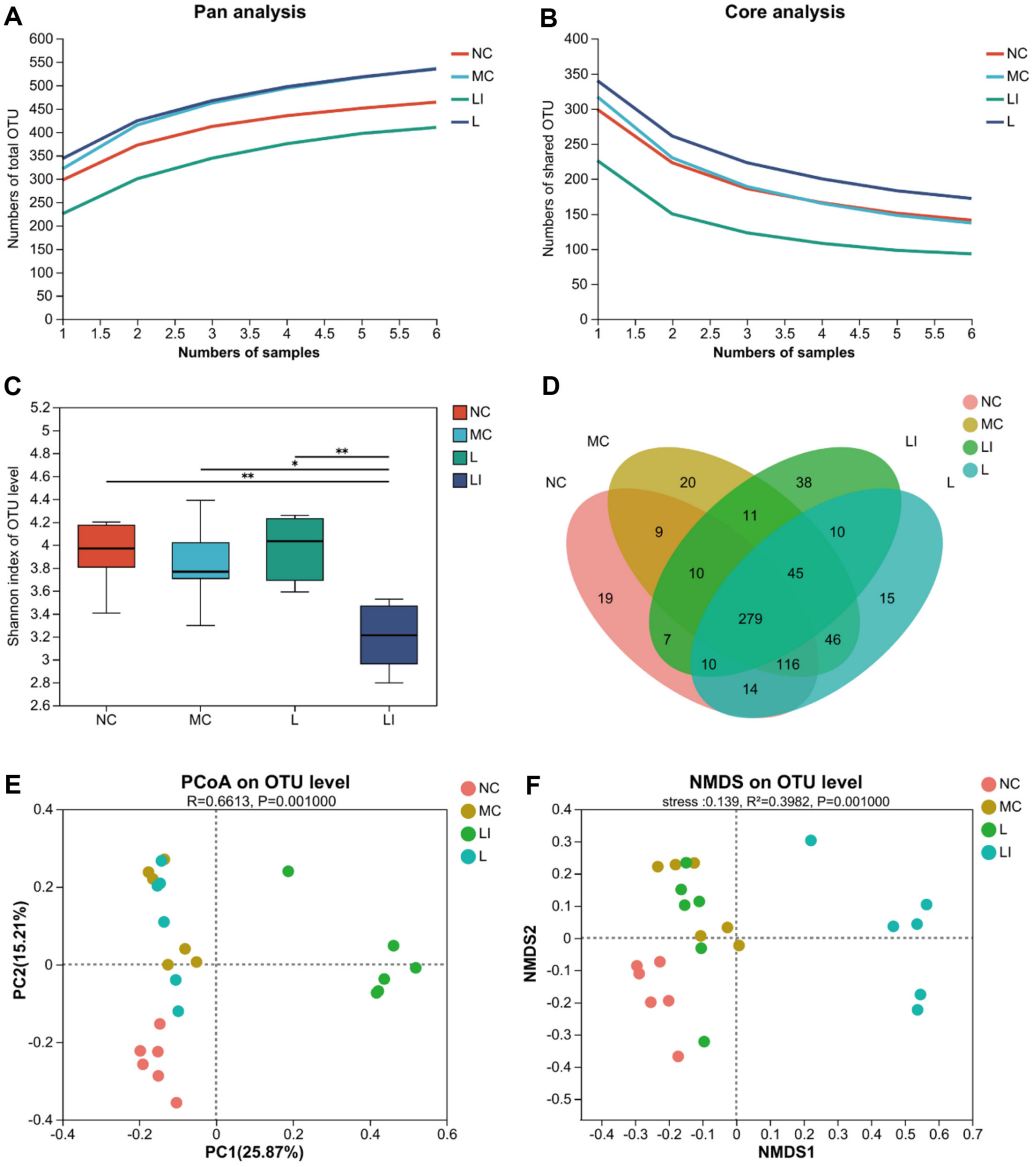
Effect of treatment intervention on gut microbiota composition. (**A**) The total number of OTU was revealed by pan analysis. (**B**) Core analysis showed that the number of core OTU. (**C**) Shannon index. (**D**) Venn diagram of OTUs in various groups. (**E**) PCoA plot based on UniFrac metrics. (**F**) NMDS revealed a difference in species in fecal samples.

The bacterial communities in the feces of mice were examined by 16S rRNA sequencing. There were 875468 sequences with a base length of 367136242 bp and an average length of 419.36 bp. OTU (similarity > 97%) analysis revealed 649 total OTUs, including 13 phyla, 18 classes, 45 orders, 77 families, 163 genera, and 258 species. Pan analysis (Fig. 2A) and core analysis (Fig. 2B) show that curves gradually smooth out, demonstrating that the sample size of this sequencing analysis was adequate and the sequencing structure could be guaranteed. The Shannon index (Fig. 2C) which reflected the α-diversity was not significantly different among the NC, MC, and L groups, but the LI group had a significantly lower Shannon index than the other three groups. Venn diagrams (Fig. 2D) showed the overlap and different numbers of OTUs among the different groups. There were 279 OTUs shared among the NC, MC, L, and LI groups, whereas there were unique OTUs in the NC (19), MC (20), L (15), and LI (38) groups, which indicated that there were some similarities and differences in microbial flora among the four groups. PCoA (Fig. 2E) and NMDS (Fig. 2F) were used for analyzing the entire bacterial community to determine the β-diversity of the gut microbiota among the four groups, which showed that the structure of the microbial community in the LI, MC, and NC groups was distinguished, while the composition of gut microbiota in the L group and MC group had some similarities, but also differences. The distance between the LI group and other groups indicated that the composition of gut microbiota was more significantly altered after treatment with humans *Lactobacillaceae* and inulin.

**Fig. 3:**
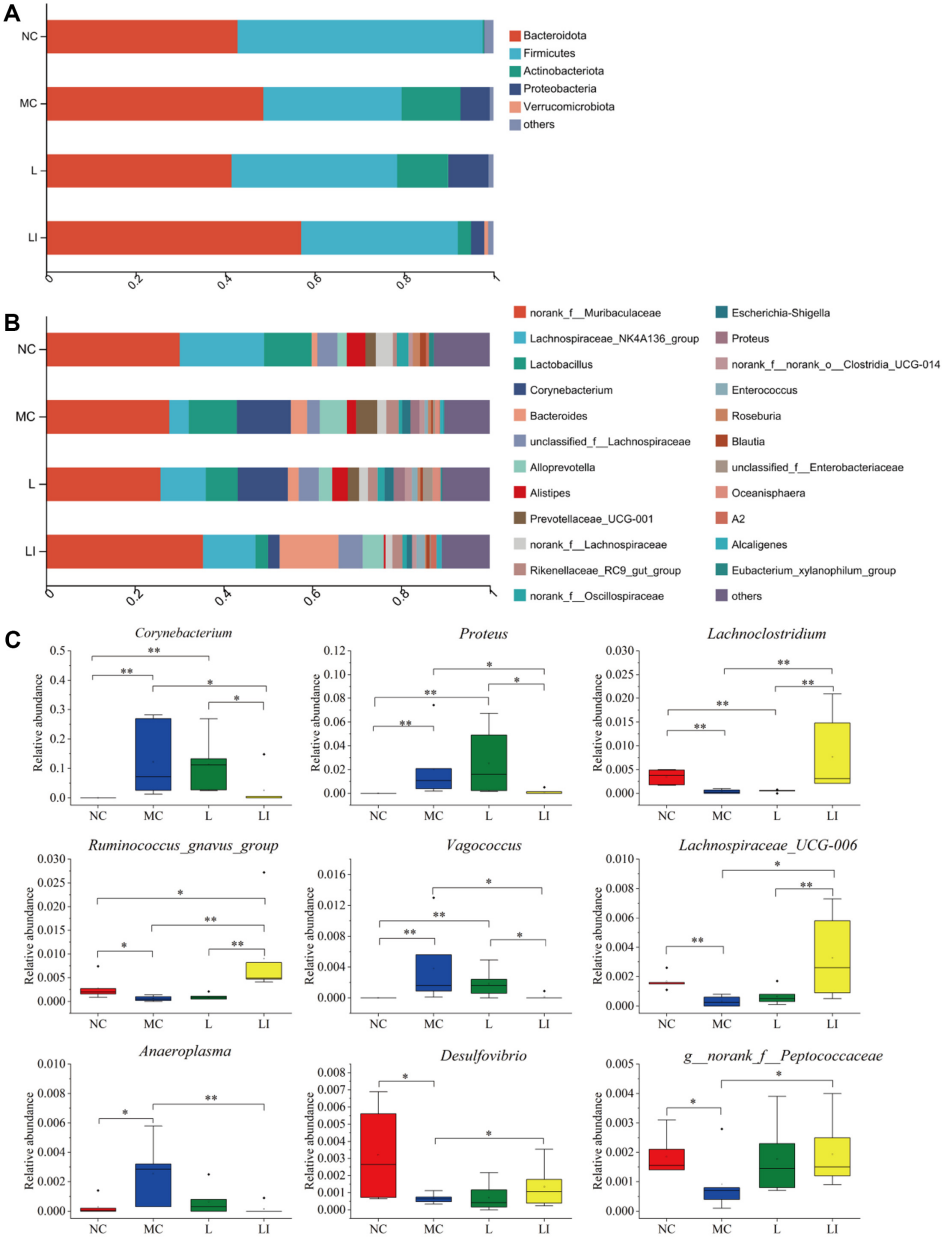
Effect of treatment on the composition of the gut microbiota in db/db mice. (**A**) Relative abundance of microbes at the phylum level. (**B**) Relative abundance of microbes at the genus level. (**C**) Changes in the relative abundance of individuals among groups. *Indicates statistical significance at *p* < 0.05. **Indicates statistical significance at *p* < 0.01.

As shown in Fig. 3A, *Firmicutes*, *Bacteroidota*, *Actinobacteriota*, and *Proteobacteria* constituted the four dominant phyla in the four groups. *Bacteroidota* and *Firmicutes* accounted for 42.79% and 54.76%, 48.55% and 30.94%, 41.42% and 37.03%, 57.01%, and 35.01% in the NC, MC, L, and LI groups, respectively, while *Actinobacteriota* and *Proteobacteria* accounted for 0.34% and 0.10%, 13.11% and 6.50%, 11.40% and 9.02%, and 2.95% and 2.96% in the four groups, respectively.

*norank_ f_ Muribaculaceae*, *Lachnospiraceae_NK4A136_ group*, and *Lactobacillus* were the three dominant genera in the four groups, as shown in Fig. 3B. The abundance of *Lachnospiraceae_NK4A136_ group* in the four groups was 18.97% (NC), 4.38% (MC), 10.21% (L), and 11.84% (LI). The abundance of *Lachnospiraceae_NK4A136_ group* in the MC group was significantly lower than in the other three groups. *Lactobacillales* were the third most common bacterial in each group, with the abundances of 10.75% (NC), 10.82% (MC), 7.24% (L), and 2.84% (LI). The abundance of *Lactobacillus* in the LI group was significantly lower than that in the other three groups. Potential differences in the genus level of gut microbiota among the four groups were investigated by Metastat analysis (Fig. 3C). 22 genera differed between the NC and MC groups, of which 9 genera were significantly altered in the LI group compared to the MC group. *Corynebacterium*, *Proteus*, *Vagococcus*, and *Anaeroplasma* were significantly enriched in the MC group compared with the NC group. Compared with the MC group, the abundance of gut microbiota in the LI group showed a significant decrease in *Corynebacterium*, *Proteus*, *Vagococcus*, and *Anaeroplasma*, and a significant increase in *Lachnoclostridium*, *Ruminococcus_gnavus_group*, *Lachnospiraceae_UCG-006*, *Desulfovibrio*, and *norank_f_Peptococcaceae*, but only one genus (*Paenalcaligenes*) significantly increased in the L group. Compared with the L group, the abundance of *Corynebacterium*, *Proteus*, and *Vagococcus* was significantly decreased, and the abundance of *Lachnoclostridium*, *Ruminococcus_gnavus_group*, and *Lachnospiraceae_UCG-006* were significantly enriched in the LI group.

### Analysis of Metabolite Profiles

**Fig. 4:**
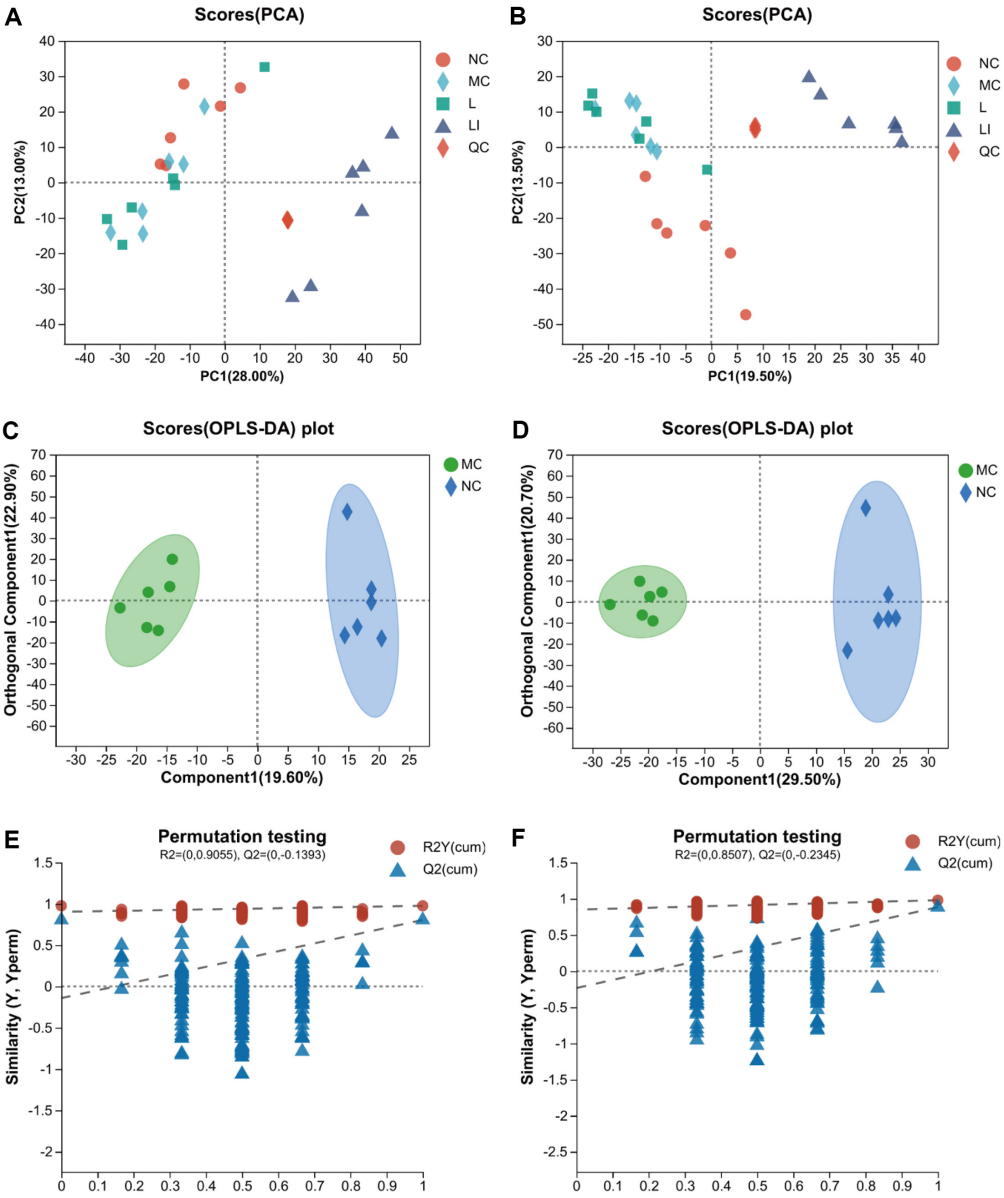
Effects of L and LI on cecum contents metabolites profiles. PCA score plots for the MC, NC, L and LI groups in posi tive (**A**) and negative (**B**) ion modes. OPLS-DA score plots between MC and NC groups in the positive (**C**) and negative (**D**) ion modes. Permutation test in positive (**E**) and negat ive (**F**) ion modes of the NC vs. MC gro up s. The x-a xis repr esents th e rep laceme nt r ete ntio n of th e rep lacement tes t.

UHPLC-MS was employed to analyze the metabolomics of cecum samples in both positive and negative ion modes, in order to investigate the effects of L and LI on intestinal metabolites in diabetic mice. PCA score plots were used to display the sample distribution and group dispersion (Fig. 4A and 4B). The results revealed a clear separation of the LI group from the other three groups in both ion modes, indicating a significant change in metabolic patterns of T2DM mice following LI treatment. The OPLS-DA (Fig. 4C and 4D) analysis identified distinct metabolites between the NC and MC groups. The models were deemed reliable based on the R2 and Q2 values of 0.978 and 0.807 for positive-ion mode, and 0.981 and 0.885 for negative-ion mode, and the absence of overfitting as demonstrated by the permutation tests (Fig. 4E and 4F). The OPLS-DA score plots clearly distinguished the MC group from the NC group in both ion modes, indicating that T2DM significantly altered the metabolic profile of healthy mice.

**Fig. 5:**
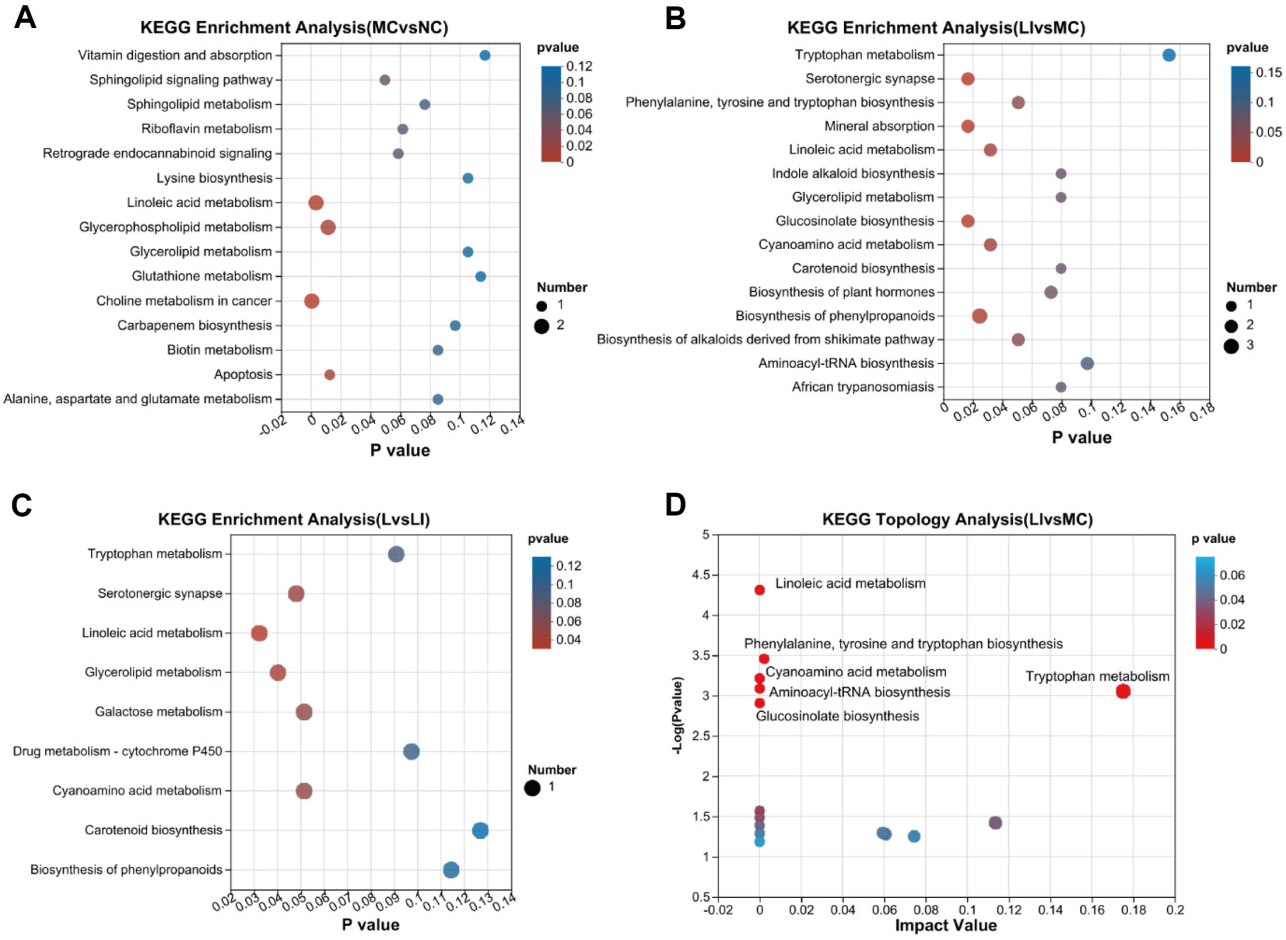
The metabolic pathways analysis. The KEGG enrichment analysis of differential metabolites (**A**) MC VS. NC. (**B**) LI VS. MC. (**C**) LI VS. L. (**D**) The bubble chart of KEGG pathway topology analysis of LI VS. MC differential metabolites.

A total of 138 distinct metabolites between the NC and MC groups were identified as potential biomarkers in both positive-ion and negative-ion modes (VIP > 1 and *p* < 0.05). Among these metabolites, 32 metabolites were in KEGG databases (Table 2). Out of the 32 metabolites, 21 metabolites were found to be significantly different between the LI and MC groups. In the L group, the levels of oryzalexin E and β-thujaplicin were found to be lower than those in the MC group. Compared with the NC group, L-phenylalanine and L-tryptophan significantly decreased in the MC group, but significantly increased by 7.61% and 11.89% in the LI group, respectively. Compared with the NC group, sphingosines (such as 3-ketosphingosine and glucosylsphingosine) were significantly decreased in the MC group, and returned to normal control (NC) levels following the LI treatment. Furthermore, the LI group exhibited a significant reduction of several metabolites that were significantly increased in the MC group compared to the NC group, including galactosylglycerol, OXYQUINOLINE, cucurbic acid, (10)-gingerol, lumichrome, lotaustralin, 1h-indole-3-carboxaldehyde, phaseolic acid, 5-hydroxyvalproic acid, dihydrophaseic acid, 13(S)-HpODE, yangonin, b-thujaplicin, 5-hydroxyindoleacetic acid, and methylsuccinic acid. Metabolic pathways in which differential metabolites were involved were identified using KEGG enrichment analysis. Comparison of the NC and MC groups (Fig. 5A) revealed that T2DM induced significant changes in metabolites in several pathways, including "choline metabolism in cancer" (*p* < 0.001), "linoleic acid metabolism" (*p* < 0.01), "glycerophospholipid metabolism" (*p* < 0.05), "apoptosis" (*p* < 0.05) and "sphingolipid signaling pathway" (*p* < 0.05). Comparison of the LI group with the MC group (Fig. 5B) showed that LI caused significant alterations in several pathways including "cyanoamino acid metabolism" (*p* < 0.05), "linoleic acid metabolism" (*p* < 0.05), "mineral absorption" (*p* < 0.05), "glucosinolate biosynthesis" (*p* < 0.05), "serotonergic synapse" (*p* < 0.05), and "biosynthesis of phenylpropanoids" (*p* < 0.05). Comparison of the L and LI groups (Fig. 5C) revealed that differential metabolites were mainly significantly enriched in "serotonergic synapse" (*p* < 0.05), "glycerolipid metabolism" (*p* < 0.05), and "linoleic acid metabolism" (*p* < 0.05) pathways. It can be inferred that the metabolic pathways of "linoleic acid metabolism" and "serotonergic synapse" may be important targets for further attention. The bubble diagram of the KEGG pathway topology (Fig. 5D) revealed that "tryptophan metabolism" was most significantly influenced by its differential metabolites between the LI and MC groups. The analysis revealed that LI treatment led to significant changes in the levels of multiple metabolites, which were found to be significantly enriched in several pathways including "serotonergic synapse", "linoleic acid metabolism", and "tryptophan metabolism". These findings suggest that LI treatment may have a beneficial effect on metabolic pathways related to "serotonergic synapse", "linoleic acid metabolism", and "tryptophan metabolism".

### Correlation between Intestinal Metabolites and Gut Microbiota

**Fig. 6:**
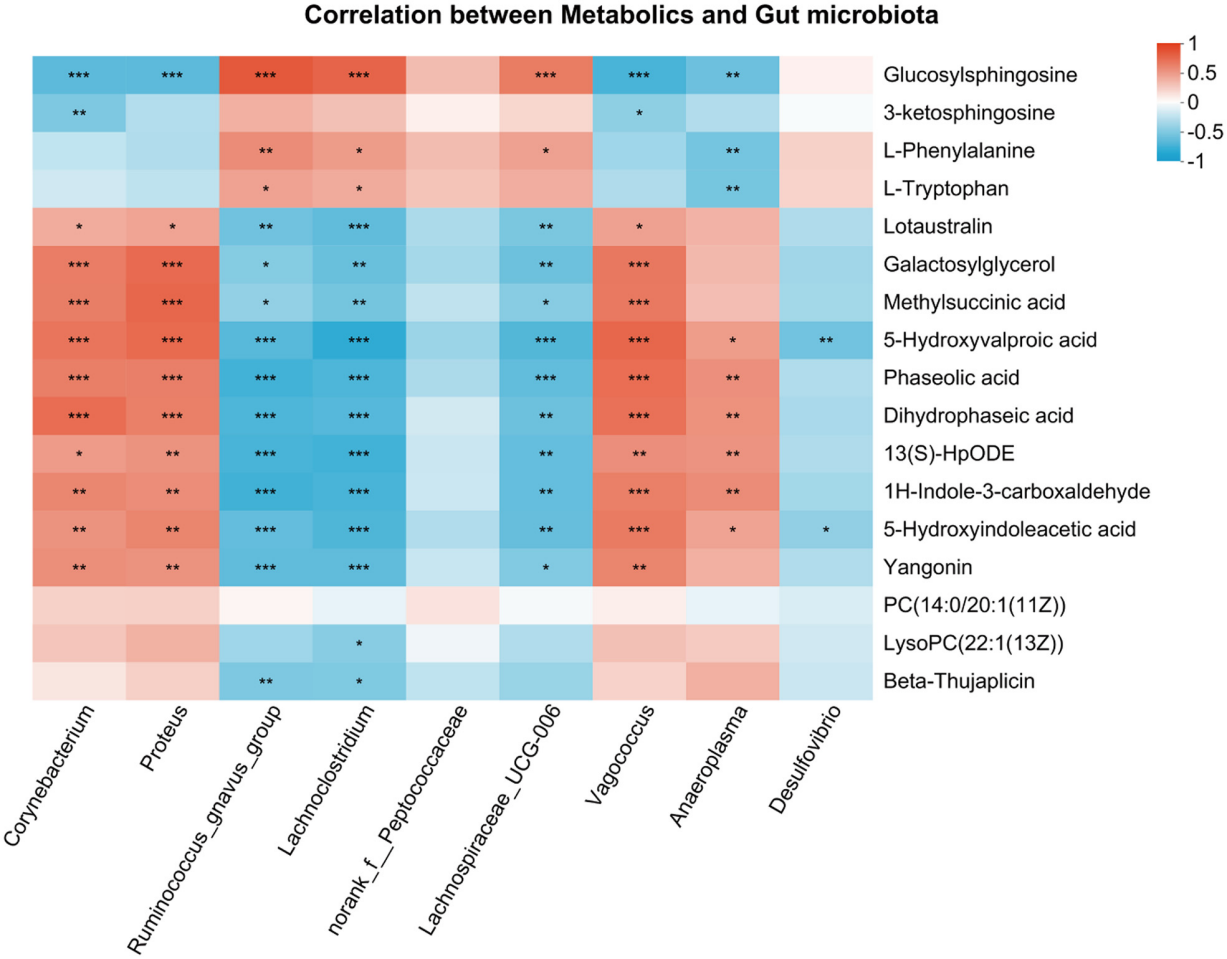
Correlation of differential metabolites and gut microbiota between LI and MC in Spearman’s correlation.

Differential OTUs of gut bacteria and metabolites between the LI and MC groups were used to establish a Spearman's correlation network (Fig. 6). The results showed that *Lachnoclostridium*, *Ruminococcus_gnavus_group*, and *Lachnospiraceae_UCG-006* were negatively correlated with 5-hydroxyvalproic acid, methylsuccinic acid, lotaustralin, yangonin, dihydrophaseic acid, phaseolic acid, 1h-indole-3-carboxaldehyde, galactosylglycerol, 5-hydroxyindoleacetic acid, and 13(S)-HpODE, while these metabolites were positively correlated with *Corynebacterium*, *Proteus*, and *Vagococcus*. L-phenylalanine and L-tryptophan were negatively correlated with *Anaeroplasma* and positively correlated with *Lachnoclostridium* and *Ruminococcus_gnavus_group*. *Desulfovibrio* was negatively correlated with 5-hydroxyvalproic acid and 5-hydroxyindoleacetic acid. PC (14:0/20:1(11Z)) and 13(S)-HpODE were involved in the "linoleic acid metabolism". L-tryptophan and 5-hydroxyindoleacetic acid were involved in "tryptophan metabolism" and "serotonergic synapse".

## Discussion

Previous research has suggested that inulin may be helpful as a supplement to treat diabetes [23]. In addition, after comparison with lycium barbarum polysaccharides (LBP) in treating T2DM rats, inulin was found to be more effective than LBP in anti-hyperglycemia and anti-inflammatory action, as well as improving both contents of SCFAs and gut barrier [24]. Inulin can improve glucolipid metabolism by suppressing mitogen-activated protein kinase and c-Jun amino-terminal kinase pathways in T2DM rats [25]. Futhermore inulin has the ability to selectively promote the growth and activity of intestinal bacteria such as *L. plantarum* [26]. *L. plantarum* can also utilize inulin to enhance its growth and improve its viability [27, 28]. In addition, a study has shown that co-administration of *L. plantarum* Lp900 with inulin can enhance the *L. plantarum* survival and persistence of the bacteria in the intestinal tracts [29]. *L. plantarum* and inulin can exert antidiabetic and antioxidant properties via improving insulin resistance and hyperlipidemia as well as hypothalamic levels of insulin, leptin, and oxidative markers in T2DM rats, and their combined recovery effect is superior to that of their use alone [30]. Therefore, we hypothesized that the combination of inulin with *L. fermentum* and *L. plantarum* might have better antidiabetic effects than either treatment alone. There is growing evidence that *L. fermentum* and *L. plantarum* may benefit human health [13, 31], and human-origin strains are preferable [32]. In this study, we found that T2DM mice had higher levels of blood glucose compared with the NC group. However, after 12 weeks of intervention with L and LI, the blood glucose levels significantly decreased and the colon morphological damage was alleviated. We found that the coordination of inulin and human-derived *Lactobacillaceae* (*L. plantarum* ZLT22, ZLT25 and *L. fermentum* ZLT11, ZLT305) had anti-diabetic effects.

Numerous studies have shown that metabolic illnesses like diabetes are linked to changes in the equilibrium between the host and gut bacteria [33, 34]. Therefore, we analyzed the composition of the gut microbiota in NC, MC, L, and LI groups by 16S rRNA sequencing. *Bacteroidetes*, *Firmicutes*, *Actinobacteria*, and *Proteobacteria* dominated the four groups, consistent with previous reports [35]. Although the relative abundance of *Firmicutes* increased in the LI and L groups, while the relative of *Bacteroidetes* increased in the LI group and decreased in the L group compared to the MC group, these changes were not statistically significant. *Verrucomicrobiota*, which is a beneficial bacteria found in healthy individuals, has shown potential in treating T2DM and related complications [6, 36, 37]. Treatment with *L. plantarum* LRCC5314 for T2DM increased the abundance of *Verrucomicrobiota* [38] which was only found in the LI group, suggesting that *Verrucomicrobiota* may be a contributing factor to the anti-diabetic effect of LI.

Compared with the MC group, the abundance of *Paenalcaligenes* was upregulated in the L group. *Paenalcaligenes* is reported to be a potential propionic acid producer [39]. In the LI group, the abundance of *Lachnospiraceae NK4A136* group, *Ruminococcus_gnavus_group*, *Lachnoclostridium*, *Desulfovibrio* *norank_f_Peptococcaceae*, and *Lachnospiraceae_UCG-006* were up-regulated, and the possible pathogenic bacteria such as *Corynebacterium* and *Proteus* were down-regulated compared with MC group. It is noteworthy that the abundance of *Lactobacillus* was more abundant in the MC group, but its abundance decreased after the L and LI interventions. Although *Lactobacillus* in the human gut is typically considered beneficial, there are some reports that an increased abundance of *Lactobacillus* has been observed in the gut microbiota of individuals with some diseases, *e.g.*, Type 2 Diabetes [40] and obesity [41]. This is similar to our result of the MC group. In addition, an investigation of the prevalence and abundance of *Lactobacillus* in 6145 individuals showed that higher abundance of *Lactobacillus* was observed in the T2DM population, and that an increased abundance of *Lactobacillus* may be associated with an increased abundance of *L. amylovorus* species [42]. Similar to our results of the L and LI groups, the higher abundance of *Lactobacillus* in T2DM rats significantly decreased after treatment with feruloylated oligosaccharides or ferulic acid [43], and the decrease in *Lactobacillus* is correlated with improvements in oral glucose tolerance and insulin sensitivity [44]. Both *Lachnospiraceae NK4A136* group and *Lachnospiraceae_UCG-006* belong to the *Lachnospiraceae* family which is characterized by its ability to convert polysaccharides into SCFAs. Previous studies have shown that enriching *Lachnospiraceae* with fiber polysaccharides can increase SCFA production, particularly acetate and butyrate, while reducing inflammation [45, 46]. *Desulfovibrio* can produce hydrogen (H_2_S) sulfide to activate the AKT pathway to improve insulin resistance [47]. However, overgrowth of *Desulfovibrio* can cause chronic inflammation due to excessive H_2_S production, which can disrupt the intestinal barrier [48, 49]. Compared to the NC group, the relative abundance of *Desulfovibrio* decreased in the MC group but returned to normal levels with LI treatment, indicating that LI treatment restored the relative abundance of *Desulfovibrio* without causing overgrowth, and therefore might improve insulin resistance. Previous studies have shown that an enrichment of menaquinone (Vitamin K2) metabolic pathways in patients with T2DM was associated with gram-positive *Corynebacterium* [50]. The abundance of *Corynebacterium* is known to increase in T2DM and is closely linked to glucose and lipid metabolism [51, 52]. Our study showed that *Corynebacterium* was significantly enriched in the MC group and significantly decreased after LI treatment, consistent with previous studies. These results suggest a potential relationship between the relative abundance of *Corynebacterium* and the pathogenesis of T2DM [53]. *Proteus* is a gram-negative bacterium, most of which are lipopolysaccharide (LPS) producers associated with diabetes [54]. The alleviation of morphological damage and inflammation in the colon could be related to *Proteus* decline. The relative abundance of *Corynebacterium* and *Proteus* increased in the MC group and decreased significantly in LI groups, suggesting that their relative abundance may be connected with the pathological state of T2DM. Peng *et al*. found that high doses of ginsenoside Rg1 could increase *Lachnoclostridium* growth in T2DM, which was discovered to have a positive correlation with the total content of SCFAs [55, 56]. Recent studies have also shown that *Lachnoclostridium* and *Ruminococcus* can increase the production of SCFAs and expression of G protein-coupled receptors (GPCRs) [57]. Both *Lachnoclostridium* and *Ruminococcus_gnavus_group* increased significantly in the LI group compared with the MC group, which suggested that LI could be useful in treating T2DM via SCFAs. Clinical studies have shown that SCFAs can help with the treatment of type 2 diabetes [58]. SCFAs have anti-inflammatory and immunomodulatory properties that may be attributed to the activation of particular cellular GPCRs [24].

One of the characteristics of diabetes is metabolic disorder [1]. Compared with the NC group, L-phenylalanine and L-tryptophan were decreased significantly in the MC group and recovered in the LI group. L-phenylalanine can regulate glucose tolerance and suppress food intake by stimulating calcium-sensing receptors to release the hormones peptide YY and glucagon-like peptide-1, and by reducing plasma ghrelin levels [59]. Diabetes causes significant depletion and loss of L-tryptophan [60]. The lower fasting glucose in the LI group might be related to the recovery of L-phenylalanine and L-tryptophan. Lotaustralin, which belongs to α-hydroxynitrile glucosides, releases hydrogen cyanide upon cleavage by β-glucosidases [61]. It has been suggested that cyanide might have a toxic influence on the pancreas, thereby precipitating diabetes [62]. Our study showed that levels of lotaustralin were elevated significantly in the MC group but decreased significantly after treatment with LI, indicating a potential correlation between the reduction of lotaustralin and the improvement of symptoms associated with T2DM. 5-hydroxyindoleacetic acid (5-HIAA) and L-tryptophan are involved in the "serotonergic synapse" and "tryptophan metabolism" pathways. "tryptophan metabolism" is the upstream metabolic pathway of "serotonergic synapse". In T2DM patients, the "serotonergic synapse" pathway is activated [63]. Our results showed that the level of 5-HIAA (a metabolite of serotonin) increased and the level of L-tryptophan (an essential amino acid for serotonin synthesis) decreased in T2DM mice, which is consistent with previous studies [64, 65]. These findings suggest that serotonin (5-HT) is synthesized from tryptophan and quickly metabolized to 5-HIAA in T2DM patients, indicating that the "tryptophan metabolism" pathway is enriched and accelerated in patients with T2DM, leading to overactivation of the "serotonergic synapse" pathway. Furthermore, the conversion of 5-HT to 5-HIAA appears to be accelerated when tryptophan levels are low. Excessive conversion of 5-HT to 5-HIAA leads to disorder of glucose and lipid metabolism [66]. 5-HT and its receptors are important regulators of islet hormone secretion, and altered actions of 5-HT contribute to β- cell dysfunction and eventual T2D [67]. 5-HT is proposed to attenuate insulin release via stimulation of Htr1a receptors, while 5-HT stimulates Htr2b receptors to increase insulin release by increasing intracellular Ca^2+^ concentration [68]. 5-HT is rapidly degraded to 5HIAA by monoamine oxidase, resulting in the failure of 5-HT to properly regulate insulin secretion [69]. Phosphatidylcholine PC(14:0/ 20:1(11Z)) and 13(S)-HpODE are involved in the "linoleic acid metabolism" pathway and were found to be significantly increased in the MC group, indicating that "linoleic acid metabolism" was disturbed in T2DM. This finding is consistent with previous studies which showed that hyperglycemia altered the "linoleic acid metabolism" pathway [70]. "linoleic acid metabolism" belongs to lipid metabolism, and lipid metabolic disorders are particularly severe in patients with insulin resistance in T2DM. Higher levels of linolenic acid (LA) in the blood were associated with a lower T2DM risk and improved insulin sensitivity [71, 72]. The elevated 13(S)-HpODE content in the MC group indicated excessive depletion of LA, leading to the enrichment of 13(S)-HpODE. Sphingosine levels decreased sharply in the T2DM group [73], which is consistent with our result. However, the decrease in sphingosine levels was reversed in the LI group. T2DM can induce abnormalities in sphingolipid metabolism and are closely associated with insulin-related diseases [74]. Sphingolipid metabolism may promote glucose 6-phosphate metabolism by upregulating sphingosine to promote the TCA cycle [73]. Our results revealed that LI could improve sphingolipid metabolism and increase blood glucose utilization, indicating an alleviating effect on diabetes.

In our study, the correlation analysis between the gut microbiota and metabolite indicators revealed that 5-hydroxyindoleacetic acid (5-HIAA) was negatively correlated with *Ruminococcus_gnavus_group*, *Lachnoclostridium*, and *Lachnospiraceae_UCG-006*. L-tryptophan was positively correlated with *Ruminococcus_gnavus_group* and *Lachnoclostridium*. L-tryptophan and 5-HIAA were involved in "serotonergic synapse" and "tryptophan metabolism". In addition, *Ruminococcus_gnavus_group* can metabolize L-tryptophan to tryptamine [75], which may limit "tryptophan metabolism" and prevent acute tryptophan depletion [76]. Compared with the MC group, LI significantly increased the abundance of *Ruminococcus_gnavus_group*, *Lachnoclostridium* and *Lachnospiraceae_UCG-006*. Therefore, LI might regulate "serotonergic synapse" and "tryptophan metabolism" by mediating *Ruminococcus_gnavus_group*, *Lachnoclostridium*, and *Lachnospiraceae_UCG-006*. Compared with the MC group, LI decreased 13(S)-HpODE significantly. 13(S)-HpODE was negatively correlated with *Ruminococcus_gnavus_group*, *Lachnoclostridium* and *Lachnospiraceae_UCG-006*. 13(S)-HpODE was involved in "linoleic acid metabolism". LI might regulate "linoleic acid metabolism" by mediating *Ruminococcus_gnavus_group*, *Lachnoclostridium*, and *Lachnospiraceae_UCG-006*.

In general, the synergism of human *Lactobacillaceae* and inulin could reduce hyperglycemia by modulating the composition of gut microbiota and regulating the metabolic profile of the intestine. Specifically, the synergism of human *Lactobacillaceae* and inulin had the potential for intestinal protection as well as anti-inflammatory action by increasing the abundance of beneficial intestinal microbes (*Verrucomicrobiota*, *Lachnospiraceae NK4A136*, *Lachnospiraceae_UCG-006*, *Lachnoclostridium*, *Ruminococcus_gnavus_group*, and *Desulfovibrio*) and decreasing the abundance of harmful intestinal microbes (*Proteus* and *Corynebacterium*), as well as providing abundant SCFAs. Moreover, the correlation analysis of gut microbiota and metabolites showed that 5-HIAA and 13(S)-HpODE were negatively correlated with *Lachnoclostridium*, *Ruminococcus_gnavus_group*, and *Lachnospiraceae_UCG-006*, but positively correlated with *Corynebacterium* and *Proteus*. L-phenylalanine and L-tryptophan were positively correlated with *Lachnoclostridium* and *Ruminococcus_gnavus_group*. In addition, the synergism of human *Lactobacillaceae* and inulin might regulate "linoleic acid metabolism", "serotonin synapse", and "tryptophan metabolism" by mediating the abundance of *Ruminococcus_gnavus_group*, *Lachnoclostridium*, and *Lachnospiraceae_UCG-006*. These results indicated that the combination of *L. plantarum* ZLT22, ZLT25 and *L. fermentum* ZLT11, ZLT305 with inulin is a promising product in the management of type 2 diabetes mellitus.

## Figures and Tables

**Table 1:** SCFA values of mice in each group.

SCFA values (μg/g)	NC	MC	L	LI
Acetic acid	316.78 ± 10.42^a^	113.41 ± 18.15^d^	156.69 ± 4.48^c^	188.62 ± 12.02^b^
Propionic acid	95.91 ± 5.61^a^	35.13 ± 2.50^c^	44.73 ± 3.53^bc^	49.15 ± 0.28^b^
Butyric acid	62.56 ± 2.09^a^	24.85 ± 1.63^c^	31.54 ± 0.31^b^	33.73 ± 0.78^b^

Experimental results using mixed linear regression models to analyze independent effects. Data are given as mean ± SD (*n* = 5),

^a, b, and c^ mean values with different letters are significantly different from each other (*p* < 0.05).

**Table 2:** The 32 differential metabolites associated with risk of T2DM in mice.

Ion modes	Metabolite	KEGG ID	MC/NC	L/MC	LI/MC	L/NC	LI/NC	Formula
Positive	Galactosylglycerol	C05401	↑ **	↑	↓^#^	↑ ^++^	↑	C_9_H_18_O_8_
	OXYQUINOLINE	C19434	↑ *	↑	↓^###^	↑ ^+^	↓ ^&^	C_9_H_7_NO
	Palmitoyl Ethanolamide	C16512	↓ *	↑	↓^#^	↓	↓^&&&^	C_18_H_37_NO_2_
	Cucurbic acid	C08482	↑ *	↑	↓^###^	↑ ^++^	↓ ^&^	C_12_H_20_O_3_
	Glucosylsphingosine	C03108	↓ **	↓	↑^###^	↓ ^++^	↑	C_24_H_47_NO_7_
	Gamma-Tocotrienol	C14155	↓ *	↑	↓	↓^+^	↓ ^&^	C_28_H_42_O_2_
	(10)-Gingerol	C17496	↑ **	↑	↓^###^	↑ ^+++^	↓ ^&&&^	C_21_H_34_O_4_
	3-ketosphingosine	C06121	↓ **	↑	↑	↓^+^	↓	C_18_H_35_NO_2_
	Lumichrome	C01727	↑ ***	↓	↓^##^	↑ ^++^	↑ ^&^	C12H10N4O_2_
	D-Biotin	C00120	↑ **	↓	↓	↑^+^	↑	C_10_H_16_N_2_O_3_S
	Lotaustralin	C08334	↑ *	↓	↓ ^###^	↑ ^+^	↓ ^&&^	C_11_H_19_NO_6_
	1-Carbapen-2-em-3-carboxylic acid	C06669	↑ ***	↑	↓	↑^+++^	↑	C_7_H_7_NO_3_
	Phenyllactic acid	C01479	↑ **	↑	↓	↑^+++^	↑	C_9_H_10_O_3_
Negative	Malic acid	C00711	↑ *	↑	↑	↑^+^	↑ ^&^	C_4_H_6_O_5_
	1H-Indole-3-carboxaldehyde	C08493	↑ ***	↑	↓^###^	↑ ^+++^	↓ ^&&&^	C_9_H_7_NO
	Phaseolic acid	C10483	↑ ***	↑	↓^###^	↑ ^++^	↓ ^&&&^	C_12_H_22_O_6_
	5-Hydroxyvalproic acid	C16650	↑ ***	↑	↓^###^	↑ ^+++^	↑ ^&^	C_8_H_16_O_3_
	Jasmonic acid	C08491	↑ *	↓	↓	↑	↑	C_12_H_18_O_3_
	Dihydrophaseic acid	C15971	↑ **	↑	↓^##^	↑ ^++^	↓	C_15_H_22_O_5_
	13(S)-HpODE	C04717	↑ **	↓	↓^###^	↑ ^+^	↓	C_18_H_32_O_4_
	PC(14:0/20:1(11Z))	C00157	↑ *	↓	↑^#^	↑ ^+^	↑ ^&&&^	C_42_H_82_NO_8_P
	LysoPC(22:1(13Z))	C04230	↑ **	↓	↓	↑^++^	↑	C_30_H_60_NO_7_P
	Oryzalexin E	C21561	↑ ***	↓ ^	↓	↑^+++^	↑	C_20_H_32_O_2_
	Sebacic acid	C08277	↑ ***	↓	↑	↑^+^	↑ ^&&^	C_10_H_18_O_4_
	Yangonin	C09980	↑ *	↑	↓^###^	↑ ^+++^	↓ ^&&&^	C_15_H_14_O_4_
	β-Thujaplicin	C09904	↑ **	↓ ^	↓ ^###^	↓	↓^&&&^	C_10_H_12_O_2_
	5-Hydroxyindoleacetic acid	C05635	↑ *	↑	↓^###^	↑ ^+^	↓	C_10_H_9_NO_3_
	Methylsuccinic acid	C08645	↑ **	↓	↓^#^	↑ ^+^	↑	C_5_H_8_O_4_
	N-acetylaspartate	C01042	↑ **	↓	↑	↑	↑^&&^	C_6_H_9_NO_5_
	Succinic acid	C00042	↑ **	↑	↑^#^	↑ ^+++^	↑ ^&&&^	C_4_H_6_O_4_
	L-phenylalanine	C00079	↓ *	↓	↑^###^	↓	↑^&&^	C_9_H_11_NO_2_
	L-tryptophan	C00078	↓ *	↑	↑^###^	↓	↑^&^	C_11_H_12_N_2_O_2_

The ↑ and ↓ in this table indicate up-regulation and down-regulation of metabolites. *p < 0.05, **p < 0.01, ***p < 0.001 * represents significant difference between NC and MC groups; ^*p* < 0.05, ^^*p* < 0.01, ^^^*p* < 0.001, ^represents significant difference between MC and L groups ; ^#^*p* < 0.05, ^##^*p* < 0.01, ^###^*p* < 0.001, #represents significant difference between MC and LI groups; ^+^*p* < 0.05, ^++^*p* < 0.01, ^+++^*p* < 0.001, +represents significant difference between NC and L groups; ^&^*p* < 0.05, ^&&^*p* < 0.01, ^&&&^*p* < 0.001, & represents significant difference between NC and LI groups.
